# Vaccination as a Key Determinant of Influenza Disease Severity in Allogeneic Hematopoietic Stem Cell Transplant Recipients: An Observational Retrospective Study

**DOI:** 10.1093/ofid/ofaf800

**Published:** 2025-12-31

**Authors:** Clara Isabel Martínez-López, Pedro Chorão, Ariadna Pérez, Brais Lamas, Dolores Gómez, Carlos Solano de la Asunción, Jaime Sanz, Juan Carlos Hernández-Boluda, David Navarro, Juan Montoro, Carlos Solano, José Luis Piñana

**Affiliations:** Department of Hematology, Hospital Clínico Universitario de Valencia, INCLIVA Biomedical Research Institute, Valencia, Spain; Hematology Division, Hospital Universitario y Politécnico La Fe, Valencia, Spain; Department of Hematology, Hospital Clínico Universitario de Valencia, INCLIVA Biomedical Research Institute, Valencia, Spain; Hematology Division, Hospital Universitario y Politécnico La Fe, Valencia, Spain; Microbiology Service, Hospital Universitario y Politécnico La Fe, Valencia, Spain; Microbiology Service, Hospital Clínico Universitario, Valencia, Spain; Hematology Division, Hospital Universitario y Politécnico La Fe, Valencia, Spain; Department of Hematology, Hospital Clínico Universitario de Valencia, INCLIVA Biomedical Research Institute, Valencia, Spain; Department of Medicine, School of Medicine, University of Valencia, Valencia, Spain; Microbiology Service, Hospital Clínico Universitario, Valencia, Spain; Department of Microbiology, School of Medicine, University of Valencia, Valencia, Spain; Hematology Division, Hospital Universitario y Politécnico La Fe, Valencia, Spain; Department of Hematology, Hospital Clínico Universitario de Valencia, INCLIVA Biomedical Research Institute, Valencia, Spain; Department of Medicine, School of Medicine, University of Valencia, Valencia, Spain; Department of Hematology, Hospital Clínico Universitario de Valencia, INCLIVA Biomedical Research Institute, Valencia, Spain

**Keywords:** allo-HCT, community acquired respiratory virus (CARV), immunodeficiency scoring index (ISI), inactivated influenza vaccine, influenza virus

## Abstract

**Background:**

Influenza virus infection remains a major cause of morbidity in allogeneic hematopoietic stem cell transplant (allo-HCT) recipients. Vaccination is a key preventive strategy; yet, clinical evidence of its benefit in this population is limited.

**Methods:**

We conducted a retrospective, multicenter observational study including adult allo-HCT recipients (≥16 years) who developed laboratory-confirmed influenza infection between 2013 and 2023, with the aim of assessing the impact of vaccination on influenza disease severity. Vaccinated status was defined as having received the seasonal influenza vaccine during the same season and before the onset of influenza infection.

**Results:**

A total of 143 recipients with 214 influenza episodes were analyzed. The median age was 45 years (range 18–70), and 58% had acute leukemia or myeloid malignancies. Most (64.3%) received transplants from unrelated or haploidentical family donors. Overall, 48 episodes (22%) occurred after influenza vaccination. At infection onset, 52% of vaccinated recipients were profoundly immunosuppressed (within <6 months post-transplant, experiencing active graft-versus-host disease, or receiving immunosuppressors or corticosteroids). Progression to lower respiratory tract disease (LRTD) occurred in 29% of episodes. Multivariable analysis showed influenza vaccination was significantly associated with reduced LRTD risk (HR 0.18; 95% CI: 0.06–0.50; *P* = .001), while a high-risk immunodeficiency scoring index (ISI) (HR 4.71; 95% CI: 1.99–11.17; *P* = .0004) and fever at screening (HR 2.16; 95% CI: 1.51–3.08; *P* < .001) independently predicted higher LRTD risk. Vaccination was also associated with decreased hospitalization risk (OR 0.20; 95% CI: 0.05–0.57; *P* = .005); whereas, high-risk ISI was linked to higher admission risk (OR 22.86; 95% CI: 4.82–170, *P* = .0003).

**Conclusions:**

This study provides real-world evidence that seasonal influenza vaccination may reduce disease severity in allo-HCT recipients and confirms the prognostic value of the ISI for disease risk assessment.

## Teaser

Among 143 allogeneic hematopoietic stem cell transplantation recipients with influenza, vaccination independently reduced progression to lower respiratory tract disease and hospitalization. The immunodeficiency scoring index stratified risk, reinforcing vaccination and early risk assessment as central components of clinical management.

Highlights

- Seasonal influenza vaccination significantly reduces LRTD progression and hospitalization in allo-HCT recipients.
- ISI at diagnosis independently predicts LRTD progression, hospital admission and oxygen requirements.
- Fever at respiratory virus screening is a good surrogate marker of LRTD progression.

Influenza virus infection is a significant cause of morbidity and mortality among patients undergoing allogeneic hematopoietic stem cell transplantation (allo-HCT) [1–4]. During the 2009, influenza A/H1N1 pandemic and in subsequent epidemic seasons, a substantially elevated risk of severe respiratory complications has been clearly demonstrated in this population [5, 6]. Their profound immunocompromised status puts these patients at increased risk for more severe infections, including progression to lower respiratory tract, oxygen requirements, hospital, and intensive care unit (ICU) admission, bacterial pneumonia, and influenza-related mortality [2, 7].

Several guidelines and scientific societies [8–12] strongly recommend seasonal influenza vaccination in allo-HCT recipients starting at 6 months post-transplant. This aims to reduce acquisition and mitigate disease severity. However, the evidence supporting its clinical benefit remains limited, and relies primarily on a small number of retrospective observational studies [13, 14]. Multiple factors have historically hindered the execution of prospective clinical trials aimed at demonstrating the clinical efficacy of influenza vaccination in this scenario. First, challenges related to the influenza virus itself, including seasonal variability in virus circulation and frequent antigenic drift, complicate the alignment between vaccine strains and circulating viruses, which in turn may limit vaccine effectiveness. Second, factors inherently related to the allo-HCT procedure such as the overall reduced number of patients compared to other targeted populations, which limit the interest of pharmaceutical industry. Third, factors related to the immunosuppressed status, such as the suboptimal immune response to vaccination with significantly lower seroprotection rates compared with immunocompetent individuals [15, 16]. Finally, the dynamic nature of immune recovery and immunosuppression status following allo-HSCT, characterized by evolving and fluctuating levels of immune reconstitution and immunosuppression, adds further complexity in the design of clinical trials to assess the clinical benefit of vaccination in this population.

Given this context, generating evidence on the clinical benefits of preventive measures, such as seasonal influenza vaccination, in this vulnerable population is essential. This holds true even when such evidence comes from retrospective observational real-world studies, as it is crucial not only for patient counseling but, perhaps more importantly, for enabling healthcare providers to confidently and convincingly recommend annual vaccination supported by scientific data. To address this need, we conducted a retrospective multicenter study aimed at investigating the effect of seasonal inactivated influenza vaccination in the severity of influenza infection in allo-HCT recipients with laboratory-confirmed influenza infection.

## METHODS

### Study Population

We performed a retrospective, multicenter, observational study in patients ≥16 years who underwent allo-HCT and subsequently developed laboratory-confirmed influenza virus infection. Data on symptomatic influenza episodes, along with clinical, transplant-related, and microbiological information, were obtained from our community-acquired respiratory virus (CARV) registry. This registry includes allo-HCT recipients aged ≥16 years who developed symptomatic CARV infections between December 2013 and June 2023 at 2 centers in Valencia, Spain: Hospital Clínico Universitario de Valencia (HCUV) and Hospital Universitario y Politécnico La Fe (HLF), as described in detail elsewhere [17]. Eligible cases consisted of consecutive symptomatic influenza episodes occurring at any time from day 0 post-transplant onward, with documented influenza vaccination status during the same season in which the infection occurred. The study protocol was approved by the local ethics committee (reference: 2019/351), and due to its retrospective and observational design, the requirement for written informed consent was waived.

### Clinical, Microbiological, and Immunological Variables

For each episode, relevant clinical and biological data were collected including age, sex, underlying hematologic malignancy, and transplant characteristics. Data on ongoing immunosuppressive drugs, systemic corticosteroid use and dose, the presence of active graft-versus-host disease (GvHD), and prior seasonal influenza vaccination status at the time of CARV screening, were also recorded at the time of CARV screening. Variables comprising the immunodeficiency scoring index (ISI), which classified patients into low, moderate, and high-risk categories as previously described [18]. Of note, recent allo-HCT or pre-engraftment was defined as allo-HCT within 30 days prior to diagnosis or absence of engraftment at the time of infection according to the ISI.

### Vaccination Policy, Data Sources, Vaccine Compositions, and Antiviral Treatment

Annual single and standard dose of trivalent (until season 2017–2018) or quadrivalent (from season 2018–2019 onwards) inactivated influenza vaccination was recommended starting from the third month post-transplant, in the absence of moderate to severe GvHD or intensive immunosuppression, in accordance with clinical guidelines [9]. Vaccination status was available for all influenza episodes included in our registry. Information on influenza vaccination status was obtained from the recipients’ electronic medical chart which is linked to the Nominal Vaccination Registry (Registro Nominal de Vacunación, RVN) of the Valencian Community, Spain. This electronic database, managed by the local health authorities, systematically records all vaccines administered in Valencian public health care centers, capturing details such as vaccine type, date of administration, healthcare facility, and patient identifiers (https://vacunaciones.san.gva.es/es/profesionales/sistema-de-informacion-vacunal).

The composition of influenza vaccines for each season followed recommendations issued by the World Health Organization. Details of vaccine components for the seasons between 2013 and 2018 have been previously detailed elsewhere [13]. For the 2018–2019 flu season, the quadrivalent vaccine contained A/Brisbane/02/2018 (H1N1), A/Kansas/14/2017 (H3N2), B/Colorado/06/2017 (Victoria lineage), and B/Phuket/3073/2013 (Yamagata lineage) strains. For the 2019–2020 season, the vaccine contained the strains A/Brisbane/02/2018 (H1N1), A/Kansas/14/2017 (H3N2), and B/Colorado/06/2017 (B/Victoria lineage) and also included a strain analogous to B/Phuket/3073/2013 (B/Yamagata lineage). For 2020–2021, it comprised A/Rabat/2020 (H1N1), A/Hong Kong/2671/2019 (H3N2), B/Washington/02/2019 (Victoria lineage), and B/Phuket/3073/2013 (Yamagata lineage). For 2021–2022, it included A/Brisbane/02/2018 (H1N1), A/Hong Kong/2671/2019 (H3N2), B/Washington/02/2019 (Victoria lineage), and B/Phuket/3073/2013 (Yamagata lineage). For 2022–2023, the formulation contained A/Darwin/9/2021 (H1N1), A/Darwin/6/2021 (H3N2), B/Austria/1359417/2021 (Victoria lineage), and B/Phuket/3073/2013 (Yamagata lineage).

Antiviral therapy consisted of oseltamivir 75 mg/Q12h for 5–10 days administered at the clinician's discretion, either in early phase upper respiratory tract disease (URTD) or in cases of severe illness, with systematic monitoring of clinical response and disease progression.

### Definitions

URTD was defined as the presence of upper airway symptoms (eg, rhinorrhea, cough, pharyngitis, and congestion) along with influenza virus detection by polymerase chain reaction (PCR), in the absence of radiologic evidence of pulmonary involvement. Possible lower respiratory tract disease (LRTD) was defined as the presence of lower respiratory symptoms and compatible radiological findings without virologic confirmation in the lower tract but with influenza confirmed by PCR in upper respiratory tract samples. Proven LRTD required detection of influenza virus in bronchoalveolar lavage (BAL) samples, along with compatible clinical and radiologic findings following previously established criteria [19]. The date of radiological proof of pulmonary involvement was used to establish the day of LRTD progression. CARV episodes were defined based on European Conference on Infections in Leukemia guidelines [20].

An influenza season was defined as the period from October to the following October, in alignment with Spain's annual vaccination campaigns, which typically begin each year in October and continue through February of the next year. Influenza vaccination status was defined as follows: recipients were considered vaccinated in the corresponding season if they received the inactivated trivalent or quadrivalent influenza vaccine prior to the onset of influenza infection during the same influenza season. The unvaccinated group included those either not receiving the vaccine during the season or developing influenza infection before receiving it within the same season. A third category (vaccination-ineligible) comprised patients who developed influenza within the first 90 days after stem cell infusion, during which period influenza vaccination was not administered to any recipient. The earliest recorded influenza vaccination occurred on day +91 post-transplant. In recipients experiencing 2 distinct episodes during the same season caused by different influenza virus subtypes (eg, Influenza A H1N1 and Influenza B), both episodes were included and classified by vaccination status at the time of each infection.

### Diagnostic Methods

Respiratory samples were obtained by nasopharyngeal aspiration, sputum, or BAL whenever possible, depending on clinical presentation and disease course. Respiratory virus PCR testing was performed using validated commercial platforms at each center, following standardized protocols. At HCUV, the Luminex xTAG RVP Fast v1 assay (Luminex Molecular Diagnostics, Toronto, Canada) was used, while HLF employed the CLART® PneumoVir DNA array assay (Genomica, Coslada, Spain). From July 2018, HLF transitioned to the BioFire FilmArray® Respiratory Panel (BioFire Diagnostics, bioMérieux, Salt Lake City, Utah), and in June 2023 switched to the Luminex xTAG RVP Fast v1 assay.

### Study Objectives and Statistical Analysis

The primary objective was to analyze the effect of vaccination status on the risk of progression to LRTD, as well as to identify other potential LRTD-associated factors. Secondary objectives included assessing the influence of influenza vaccine status on the need for hospitalization and oxygen supplementation.

Patient characteristics were summarized using descriptive statistics based on available data. Continuous variables were reported as medians and ranges, and categorical variables as absolute frequencies and percentages. Group comparisons were performed using Fisher's exact test or median test, as appropriate. Univariable and multivariable Cox regression models were used to analyze predictors of LRTD progression. Cumulative incidence curves of LRTD progression were built to visually illustrate the effect of relevant risk factors. Logistic regression models were applied to identify independent risk factors for hospitalization and oxygen support. Variables with *P* ≤ .10 in univariable analysis were included in the multivariable models. A *P*-value < .05 was considered statistically significant. Statistical analyses were performed using SPSS (version 20.0) statistical package and R software.

## RESULTS

### Patient and Transplant Characteristics

A total of 143 allo-HCT recipients developing 214 laboratory-confirmed influenza virus infection episodes were included. The main transplant and patient characteristics are detailed in Table 1. The median age was 45 years (range: 18–70), and 57% were male. Acute leukemias, myelodysplastic syndromes, or myeloproliferative neoplasms (58%) were the most frequent diseases, followed by lymphoproliferative disorders (28.7%). Prior autologous transplant had been performed in 28.7% of the cohort. Regarding donor type, most recipients (64.3%) were allografted from an alternative donor (unrelated donor or haploidentical family donor). Peripheral blood was the predominant stem cell source (79%). Antithymocyte globulin (ATG) was administered in 25.9% of cases. The most frequent GvHD prophylaxis regimen was based on calcineurin inhibitors (53.8%), followed by post-transplant cyclophosphamide-based regimens (36.4%). The median time from allo-HCT to influenza infection was 427 days (range, 0–5329), with 25.2% of episodes occurring before day +180. In 24 cases (11.2%) influenza infection occurred before day +90 after stem cell infusion. One-hundred and sixteen patients (81%) developed 1 influenza infection episode, whereas 22 (15%) developed 2 and 5 (4%) developed 3 or more. Median follow-up for survivors after influenza infection was 1077 days (range, 100–3893).

**Table 1.** Patient and Transplant Characteristics

Characteristics	Whole Cohort (*n* = 143)
Age at allo-HCT (y), median (range)	45 (18–70)
Male, *n* (%)	82 (57)
Underlying malignancies, *n* (%)	
AL/MDS/MPN	83 (58)
Chronic myeloid leukemia	7 (4.9)
Lymphoid disorders	41 (28.7)
Plasma cell disorders	7 (4.9)
Others	5 (3.5)
Prior autologous-HCT, *n* (%)	41 (28.7)
Positive recipient CMV serology, *n* (%)	116 (81.1)
Donor female to male recipient, *n* (%)	26 (18.2)
Transplant period, *n* (%)	
≥ 2020	9 (6.3)
2017–2019	42 (29.4)
2014–2016	47 (32.9)
< 2014	45 (31.4)
Conditioning regimen, *n* (%)	
RIC	72 (50.3)
MAC	71 (49.7)
Type of donor, *n* (%)	
HLA-identical sibling	51 (35.7)
HLA-identical unrelated donor	31 (21.7)
HLA-mismatched unrelated donor	34 (23.8)
Haploidentical family	25 (17.4)
HLA-mismatched sibling	2 (1.4)
Stem cell source, *n* (%)	
PB	113 (79)
UCB	3 (2.1)
BM	27 (18.9)
ATG as a part of conditioning regimen, *n* (%)	37 (25.9)
GvHD prophylaxis, *n* (%)	
Tacrolimus and sirolimus	14 (9.8)
Post-Cy	52 (36.4)
Calcineurin inhibitors-based	77 (53.8)
Median time from allo-HCT to influenza, d (range)	427 (0–5329)
Number of influenza episodes, *n* (%)	
1	116 (81)
2	22 (15)
> 2	5 (4)
Time from allo-HCT to first influenza episode (categories), *n* (%)	
Until d +180	36 (25.2)
181–1 y	34 (23.8)
>1 y	73 (51)
All-cause mortality at d +100 after the last influenza episode, *n* (%)	10 (7)
Infectious and/or respiratory failure mortality at d +30 after the last influenza episode, *n* (%)	3 (2.1)
Infectious and/or respiratory failure mortality at d +100 after the last influenza episode, *n* (%)	6 (4.2)
Median follow-up for survivors after the last influenza episode, d (range)	1077 (100–3893)

Abbreviations: allo-HCT, allogeneic hematopoietic stem cell transplantation; AL, acute leukemia; MDS, myelodysplastic syndrome; MPN, myeloproliferative neoplasm; RIC, reduced intensity conditioning; MAC, myeloablative conditioning; PB, peripheral blood; UCB, umbilical cord blood; BM, bone marrow; HLA, human leucocyte antigen system; ATG, antithymocyte globulin; GvHD, graft versus host disease; Post-Cy, post-transplant cyclophosphamide.

### Characteristics According to Upper or Lower Respiratory Tract Involvement

**Figure 1.**
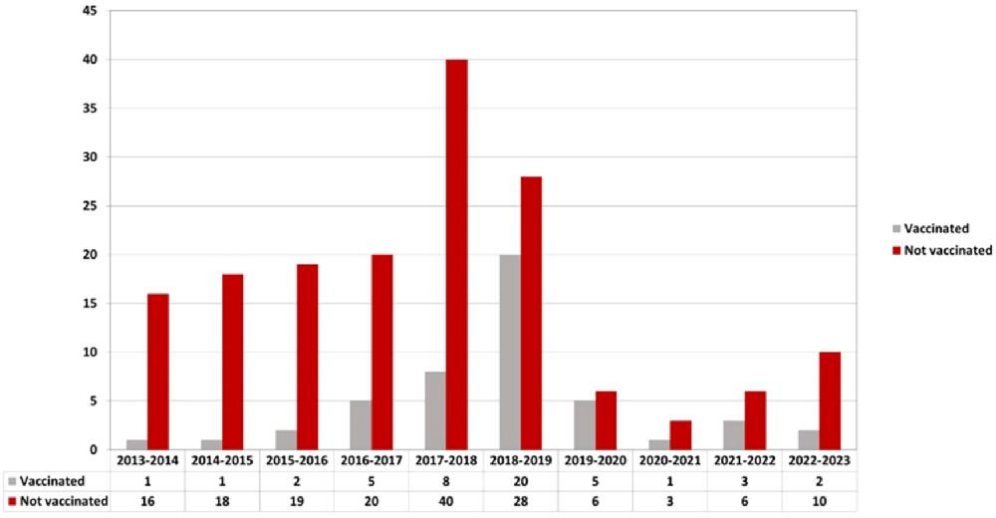
Distribution of influenza episodes by vaccination status across seasonal influenza periods from 2013–2014 to 2022–2023. This bar chart illustrates the number of laboratory-confirmed influenza episodes among allogeneic hematopoietic stem cell transplantation recipients stratified by vaccination status at each influenza season.

The 214 influenza episodes stratified by involvement of only URTD, possible or proven LRTD are summarized in Table 2. Overall, 48 (22%) out of 214 episodes occurred after seasonal influenza vaccination. Figure 1 shows the distribution of influenza infection episodes over seasons according to vaccination status. Briefly, 151 (71%) episodes were limited to URTD, whereas 63 (29%) progressed to LRTD, with 41 (19%) classified as possible and 22 (10%) as proven LRTD. Recipients with proven LRTD were significantly older at the time of transplant (median age 53 years) than those with URTD (median age 41 years) [*P* = .006]. Several immunosuppression conditions were significantly associated with proven LRTD progression, such as lymphopenia at different cutoffs (*P* < .001), concurrent corticosteroid use (*P* = .003) and active GvHD (*P* = .04). The high-risk ISI was also overrepresented in cases of proven LRTD (*P* < .001). In contrast, influenza vaccination was more common in cases limited to the URTD (*P* = .007). Coinfections (viral, bacterial, and fungal) were more common in cases of LRTD, as was the need for hospitalization (*P* < .001), oxygen therapy (*P* < .001), and ICU admission (*P* < .001).

**Table 2.** Clinical and Biological Characteristics of Influenza Infection Episodes in Allo-HCT Recipients According to Upper or Lower Respiratory Tract Involvement

	Only URTD (*n* = 151)	Progression to Possible LRTD (*n* = 41)	Progression to Proven LRTD (*n* = 22)	*P*-Value
Transplant characteristics				
Age at allo-HCT (y), median (range)	41 (10–70)	49 (26–67)	53 (26–69)	.006
ATG as part of conditioning, *n* (%)	33 (22)	9 (22)	8 (36)	.31
Positive recipient CMV serology, *n* (%)	120 (80)	30 (73)	19 (86)	.45
Donor female to male recipient, *n* (%)	35 (23)	10 (24)	4 (18)	.84
GvHD prophylaxis, *n* (%)				.59
Tacrolimus and sirolimus	19 (13)	3 (7)	3 (14)	
Post-Cy	60 (40)	13 (32)	7 (32)	
Calcineurin inhibitors-based	72 (47)	25 (61)	12 (54)	
Type of donor, *n* (%)				.62
HLA-identical sibling	51 (34)	17 (41)	9 (41)	
HLA-identical unrelated donor	31 (21)	7 (17)	3 (14)	
HLA-mismatched unrelated donor	35 (23)	7 (17)	2 (9)	
Haploidentical family	32 (21)	10 (25)	8 (36)	
HLA-mismatched sibling	2 (1)	0 (0)	0 (0)	
Donor/recipient HLA mismatch	65 (43)	17 (41)	11 (50)	.79
Immunodeficiency scoring index, *n* (%)^a^				
ANC < 0.5 × 10^9^/L	3 (2)	3 (7)	3 (14)	.01
ALC < 0.2 × 10^9^/L	4 (3)	3 (7)	6 (27)	.0003
Age ≥ 40 y	98 (65)	32 (78)	19 (86)	.05
Myeloablative conditioning regimen	66 (44)	20 (49)	10 (45)	.93
Corticosteroids	49 (32)	20 (49)	15 (68)	.0027
Recent allo-HCT or pre-engraftment	6 (4)	2 (5)	1 (5)	.87
Active GvHD	67 (44)	22 (54)	16 (73)	.04
ISI, *n* (%)				.00001
Low risk (0–2)	68 (45)	13 (32)	3 (14)	
Moderate risk (3–6)	80 (53)	25 (61)	13 (59)	
High risk (7–12)	3 (2)	3 (7)	6 (27)	
Other characteristics^a^				
On IS, *n* (%)	102 (67)	29 (71)	18 (82)	.43
ALC < 0.1 × 10^9^/L, *n* (%)	4 (3)	3 (7)	3 (14)	.02
ALC < 0.5 × 10^9^/L, *n* (%)	20 (13)	11 (27)	8 (36)	.01
ALC < 1 × 10^9^/L, *n* (%)	58 (38)	20 (49)	12 (54)	.26
RVI characteristics and clinical consequences				
Influenza type, *n* (%)				.30
A	115 (76)	28 (68)	17 (77)	
B	30 (20)	13 (32)	5 (23)	
C	6 (4)	0 (0)	0 (0)	
Oseltamivir therapy, *n* (%)	117 (77)	37 (90)	21 (95)	.08
Influenza vaccine, *n* (%)				.007
Yes	44 (29)	3 (7)	1 (5)	
No	90 (60)	33 (81)	19 (86)	
Vaccination-ineligible	17 (11)	5 (12)	2 (9)	
Respiratory coinfections^b^, *n* (%)				<.0001
Other CARVS	0 (0)	10 (25)	10 (45)	
Bacterial	0 (0)	4 (10)	2 (9)	
Fungal	1 (1)	3 (7)	3 (14)	
BAL, *n* (%)	1 (1)	7 (17)	22 (100)	<.0001
Hospital admission, *n* (%)	17 (11)	28 (68)	19 (86)	<.0001
ICU admission, *n* (%)	1 (1)	3 (7)	7 (32)	<.0001
Oxygen support, *n* (%)	3 (2)	12 (29)	12 (54)	<.0001

Abbreviations: URTD, upper respiratory tract disease; LRTD, lower respiratory tract disease; allo-HCT, allogeneic hematopoietic stem cell transplantation; ATG, antithymocyte globulin; GvHD, graft-versus-host disease; Post-Cy, post-transplant cyclophosphamide; HLA, human leukocyte antigen; ANC, absolute neutrophil count; ALC, absolute lymphocyte count; MAC, myeloablative conditioning; ISI, immunodeficiency scoring index; RVI, respiratory virus infection; IS, immunosuppressants; CARV, community acquired respiratory virus; ICU, intensive care unit; BAL, bronchoalveolar lavage.

^a^All variables were captured at the time of influenza diagnosis.

^b^Respiratory coinfections were defined by detection of a significant copathogen requiring antimicrobial intervention in concurrent nasopharyngeal or BAL samples during influenza infection and until clinical and/or microbiological resolution. Coinfections also include the detection of other CARVs in the same influenza sample.

### Comparison of Characteristics Among Vaccinated, Unvaccinated, and Vaccination-Ineligible Patients

To better interpret the potential effect of vaccination in the influenza severity, we compared the baseline characteristics of the 3 different groups (unvaccinated, vaccinated, and vaccination-ineligible). As shown in Table 3, several variables differed significantly across groups; however, these disparities were mainly driven by the vaccination-ineligible group, whose early post-transplant status explains their distinctive clinical and immunological profile (profound lymphopenia, pre-engraftment or recent allo-HCT, higher ISI scores, and clustering within the first 180 days after transplant). In contrast, vaccinated and unvaccinated patients showed broadly similar demographic and transplant-related features, with only modest differences in GVHD prophylaxis, use of immunosuppressants, presence of active GVHD at the time of infection, and ALC values. Notably, ISI scores categories were comparable between vaccinated and unvaccinated individuals. Importantly, transplant period differed significantly, reflecting a higher proportion of vaccinated cases in more recent years. All 4 vaccinated patients who developed LRTD had no GVHD, were not receiving immunosuppression, were >1 year post-allo-HCT and had ALC >1000 × 10⁹/L at diagnosis.

**Table 3.** Comparison of Characteristics Among Vaccinated, Unvaccinated, and Vaccination-ineligible Patients

	Unvaccinated (*n* = 142)	Vaccinated (*n* = 48)	*P*-Value^a^	Ineligible For Vaccination (*n* = 24)	*P*-Value^b^
Transplant characteristics					
Age at allo-HCT (y), median (range)					
Recipient CMV positive, *n* (%)	116 (82)	35 (73)	.21	18 (75)	.38
Prior HCT, *n* (%)	37 (26)	14 (29)	.7	10 (42)	.3
ATG as part of conditioning, *n* (%)	40 (28)	7 (15)	.08	3 (13)	.07
GvHD prophylaxis, *n* (%)			.001		.001
Tacrolimus and sirolimus	23 (16)	2 (4)		0	
Post-Cy	36 (25)	28 (58)		16 (67)	
Calcineurin inhibitors-based	83 (58)	18 (38)		6 (33)	
Type of donor, *n* (%)			.2		.46
HLA-identical sibling	54 (38)	14 (29)		9 (38)	
HLA-identical unrelated donor	26 (18)	13 (27)		5 (21)	
HLA-mismatched unrelated donor	38 (27)	7 (16)		5 (21)	
Haploidentical family	23 (16)	13 (27)		5 (21)	
HLA-mismatched sibling	1 (1)	1 (2)		0	
Donor/recipient HLA mismatch	62 (44)	21 (44)	.99	10 (42)	.98
Female donor to male recipient	31 (22)	11 (23)	.9	7 (29)	.73
Stem cell source, *n* (%)			.72		.82
PB	111 (78)	39 (81)		19 (79)	
UCB	27 (19)	7 (15)		5 (21)	
BM	4 (3)	2 (4)		0	
Transplant period, *n* (%)			.005		.001
≥ 2020	6 (4)	5 (10)		3 (13)	
2017–2019	32 (23)	18 (38)		11 (46)	
2014–2016	46 (32)	18 (38)		8 (33)	
< 2014	58 (41)	7 (15)		2 (8)	
Immunodeficiency scoring index, *n* (%)^c^					
ANC < 0.5 × 10^9^/L	3 (2)	1 (2)	.9	5	.001
ALC < 0.2 × 10^9^/L	5 (3.5)	0	.33	8 (33)	.001
Age ≥ 40 y	101 (71)	32 (67)	.6	16 (67)	.79
Myeloablative conditioning regimen	69 (49)	21 (44)	.61	11 (46)	.83
Corticosteroids	63 (44)	14 (29)	.06	7 (29)	.083
Recent allo-HCT or pre-engraftment	0	0		9 (38)	.001
Active GvHD	82 (57)	18 (43)	.02	5 (21)	.001
ISI, *n* (%)			.34		.001
Low risk (0–2)	54 (38)	20 (42)		10 (42)	
Moderate risk (3–6)	82 (58)	28 (58)		8 (33)	
High risk (7–12)	6 (4)	0		6 (25)	
Other characteristics^c^					
On IS, *n* (%)	101 (71)	25 (52)	.02	23 (96)	.001
ALC < 0.1 × 10^9^/L, *n* (%)	2 (1)	0	.9	8 (33)	.001
ALC < 0.5 × 10^9^/L, *n* (%)	25 (17)	2 (4)	.02	15 (63)	.001
ALC < 1 × 10^9^/L, *n* (%)	60 (42)	11(23)	.02	19 (79)	
Oseltamivir therapy, *n* (%)	104 (73)	39 (81)	.2	19 (79)	.3
LRTD, *n* (%)	52 (37)	4 (8)	.001	7 (29)	.001
Time from allo-HCT to influenza (categories), *n* (%)			.05		.001
Until d +180	21 (15)	1 (2)		24	
181–1 y	39 (27)	12 (25)		0	
>1 y	82 (58)	35 (73)		0	

Abbreviations: URTD, upper respiratory tract disease; LRTD, lower respiratory tract disease; allo-HCT, allogeneic hematopoietic stem cell transplantation; ATG, antithymocyte globulin; GvHD, graft-versus-host disease; Post-Cy, post-transplant cyclophosphamide; HLA, human leukocyte antigen; ANC, absolute neutrophil count; ALC, absolute lymphocyte count; MAC, myeloablative conditioning; ISI, immunodeficiency scoring index; RVI, respiratory virus infection; IS, immunosuppressants; CARV, community acquired respiratory virus; ICU, intensive care unit; BAL, bronchoalveolar lavage.

^a^ *P*-value refers to comparisons between the vaccinated and unvaccinated groups, excluding the ineligible group.

^b^ *P*-value refers to the comparisons among the 3 groups.

^c^All variables were captured at the time of influenza diagnosis.

### Lower Respiratory Tract Disease Progression and Risk Factors

**Figure 2.**
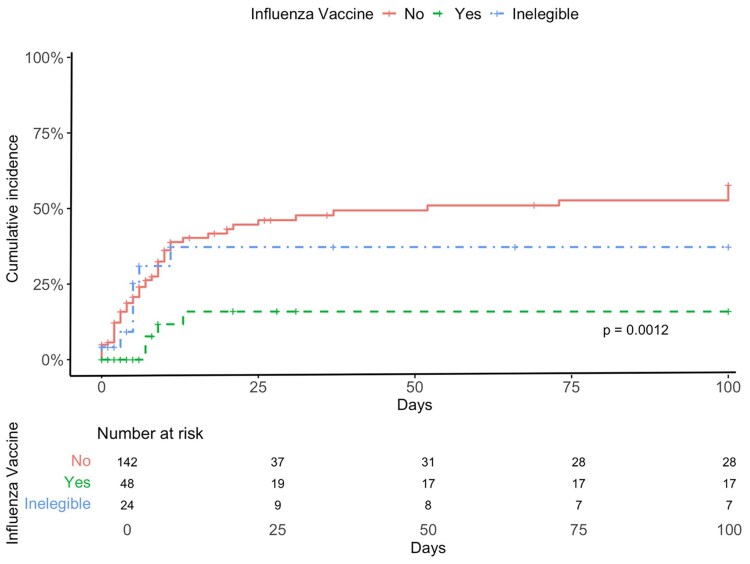
Cumulative incidence of progression to lower respiratory tract infection (LRTD) according to patient vaccination status.

**Figure 3.**
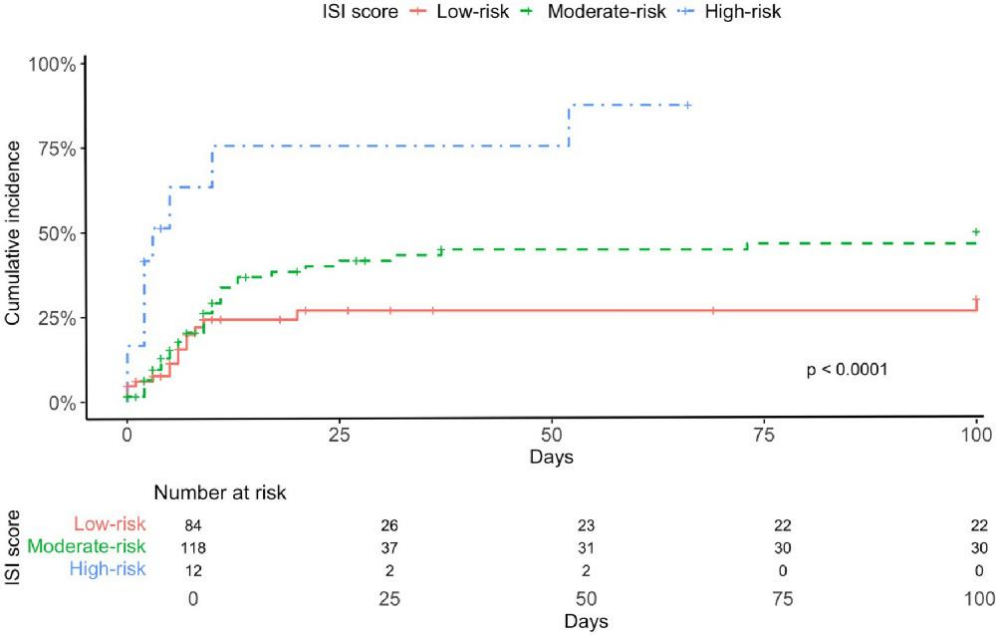
Cumulative incidence of progression to lower respiratory tract disease (LRTD) according to risk group based on ISI score.

**Figure 4.**
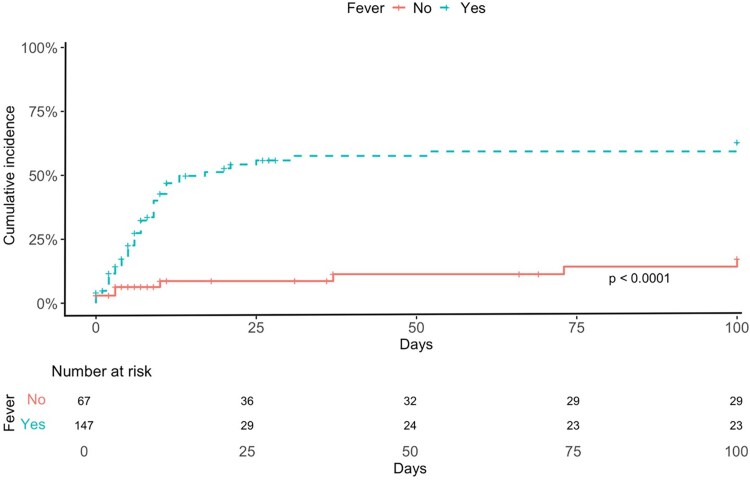
Cumulative incidence of progression to lower respiratory tract infection (LRTD) according to presence of fever at CARV screening.

Overall, LRTD progression occurred in 29% of cases at a median of 3 days (range 0–28 days) after CARV PCR screening. Table 4 shows univariable and multivariable analyses for potential risk factors for progression to LRTD. In multivariable analysis, independent risk factors were high-risk ISI (HR 4.77; *P* = .0004) and fever at the time of influenza diagnosis (HR 4.49; *P* < .001). Influenza vaccination was an independent protective factor against LRTD progression (HR 0.19; *P* = .001). Cumulative incidence curves of LRTD progression by vaccination status, ISI category and fever are shown in Figures 2–4.

**Table 4.** Univariate and Multivariate Analysis of Risk Factors for Influenza Lower Respiratory Tract Disease (LRTD) and Overall Mortality

Variables	Cox. Regression Progression to LRTD (*n* = 214)			
	Univariate Analysis		Multivariate Analysis	
	HR (95% CI)	*P*-Value	OR (95% CI)	*P*-Value
Patient CMV serology				
Positive	.93 (.51–1.70)	.835		
Negative	1			
Donor/recipient sex mismatch				
Female to male	.82 (.45–1.48)	.51		
Other combinations	1			
Recipient sex				
Male	1			
Female	1.24 (.75–2.03)	.39		
Donor/recipient HLA mismatch	.87 (.53–1.44)	.59		
ATG as a part of conditioning	1.03 (.59–1.81)	.89		
Type of donor				
HLA id and mm sibling	1			
HLA id URD	.66 (.32–1.39)	.28		
HLA mm URD	.53 (.52–1.15)	.11		
Haplo id	.79 (.43–1.45)	.46		
GVHD prophylaxis				
Sir-Tac	1			
Post-Cy	1.03 (.41–2.58)	.94		
Calcineurin inhibitors-based	1.35 (.57–3.20)	.49		
Corticosteroids	2.24 (1.35–3.69)	.00156	NT	
Corticosteroids dose				
No	1			
<30 mg/d	2.07(1.10–3.89)	.02	1.65 (.81–3.35)	.16
≥30 mg/d	2.51 (1.41–4.46)	0001	1.59 (.76–3.32)	.21
On IS	1.21 (.68–2.14)	.49		
ALC < 0.5 × 109/L	2.02 (1.18–3.47)	.01	1.05 (.51–2.16)	.88
ALC < 0.2 × 109/L	1.57 (1.24–2)	.0001	NT	
ALC <0.1 × 109/L	3.40 (1.45–7.95)	.004	NT	
ALC < 1 × 109/L	1.48 (1.12–1.97)	.005		
ANC < 0.5 × 109/L	1.48 (1.08–2.02)	.01	NT	
Age ≥ 40 y	1.40 (.84–2.32)	.19	NT	
Active GvHD at the time of RVI	1.08 (.33–3.45)	.89	NT	
Recent allo-HCT or pre-engraftment	1.48 (1.12–1.97)	.005	NT	
Time from allo-HCT to the influenza detection				
≤180 d	1			
181 to 365 d	.69 (.34–1.41)	.31		
>365 d	.75 (.75–1.32)	.35		
MAC	.98 (.59–1.62)	.94		
Transplant period				
≥ 2020	1			
2017–2019	1.78 (.41–7.58)	.43		
2014–2016	1.11 (.25–4.91)	.88		
< 2014	1.85 (.43–7.86)	.40		
Oseltamivir at the URTD stage	.90 (.54–1.48)	.68		
Influenza vaccine^a^				
No	1		1	
Yes	.18 (.06–.52)	.0012	.19 (.06–.53)	.001
Vaccination-ineligible	.67 (.3–1.48)	.32	.73 (.32–1.65)	.45
Coinfections	2.14 (1.26–3.63)	.004	1.51 (.86–2.66)	.14
Fever at the time of CARV screening	2.11 (1.48–3.01)	<.001	4.49 (2.10–9.61)	<.001
ISI				
Low risk (0–2)	1		1	
Moderate risk (3–6)	1.59 (.88–2.85)	.12	1.51 (.83–2.72)	.16
High risk (7–12)	6.35 (2.78–14.49)	.00001	4.77 (1.98–11.50)	.0004

Abbreviations: CI, confidence interval; Log. Regr, logistic regression model; OR, odds ratio; Cox Regr, cox regression; HR, hazard ratio; ATG, antithymocyte globulin; Post-Cy, post-transplant cyclophosphamide; URTD, upper respiratory tract disease; LRTD, lower respiratory tract disease; GvHD, graft-versus-host disease; allo-HCT, allogeneic hematopoietic stem cell transplantation; mm URD, mismatch unrelated donor; Haplo id, haploidentical family donor; id sibling, identical sibling donor; id URD, identical unrelated donor; UCB, umbilical cord blood; MAC, myeloablative conditioning; CARV, community acquired respiratory virus; ISI, immunodeficiency score index; ANC, absolute neutrophil count; ALC, absolute lymphocyte count; NT, not tested.

^a^A repeated multivariable analysis was performed excluding the vaccination-ineligible group, and influenza vaccination remained a significant protective factor against LRTD (HR 0.18, 95% CI .07–.51, *P* = .0011).

### Risk Factors for Hospitalization and Oxygen Therapy Requirement

As shown in Table 5, several variables were significantly associated with increased risk of hospitalization and oxygen supplementation after influenza infection in allo-HCT recipients.

**Table 5.** Univariate and Multivariate Logistic Regression Analysis of Risk Factors for Hospitalization and Oxygen Therapy Requirement in the Context of Influenza Infection

Variables	Log. Regr. For Hospital Admission (*n* = 214)				Log. Regr. For Oxygen Therapy (*n* = 214)			
	Univariate analysis		Multivariate analysis		Univariate analysis		Multivariate analysis ^a^	
	OR (95% CI).	*P*-Value	OR (95% CI)	*P*-Value	OR (95% CI)	*P*-Value	OR (95% CI)	*P*-Value
Patient CMV serology								
Positive	1.39 (.67–3.08)	.38			1.61 (.58–5.73)	.4		
Negative	1				1			
Donor/recipient sex mismatch								
Female to male	1.01 (.49–2.01)	.96			2.69 (1.13–6.24)	.02	3.24 (1.27–8.21)	.01
Other combinations	1				1			
Recipient sex								
Male	1				1			
Female	1.53 (.84–2.77)	.15			1.24 (.54–2.80)	.59		
Donor/recipient HLA mismatch	.52 (.27–.95)	.038	.32 (.12–.84)	.02	.61 (.25–1.4)	.25		
ATG as a part of conditioning	.89 (.43–1.78)	.76			.71 (.22–1.86)	.52		
Type of donor								
HLA id and mm sibling	1				1			
HLA id URD	.42 (.17–.94)	.041			.18 (.04- 1.02)	.06		
HLA mm URD	.47 (.21–1.002)	.06			.39 (.12–1.07)	.09		
Haplo id	.31 (.12-.73)	.02			.36 (.1–1.06)	.09		
GVHD prophylaxis								
Sir-Tac	1		1		1			
Post-Cy	2.36 (.71–1.74)	.19	3.68 (.72–23.01)	.13	2.30 (.38–44.1)	.44		
Calcineurin inhibitors-based	4.66 (1.49–2.57)	.01	7.04 (1.59–41.11)	.01	5.06 (.97–93.29)	.12		
Corticosteroids	4.31 (2.33–8.13)	.000004			6.77 (2.75–19.24)	.00008		
Corticosteroids dose								
No	1		1		1			
<30 mg/d	2.33 (1.07–5.03)	.03	1.4 (.51–3.6)	.31	3.90 (1.22–12.84)	.02	3.21 (.93–11.54)	.06
≥30 mg/d	8.39 (3.87–18.99)	.0000001	2.86(1.09–8.68)	.04	1.12 (4.07–34.00)	.0000062	8.21 (2.43–31.71)	.001
On IS	2.23 (1.12–4.73)	.02			2.69 (.98–9.49)	.07		
ALC < 0.5 × 109/L	4.55 (2.21–9.59)	.00004	1.97 (.7–5.3)	.19	3.14 (1.27–7.47)	.0104	.89 (.23–2.92)	.86
ALC < 0.2 × 109/L	1.28 (2.48–69.56)	0003			5.15 (1.24–19. 45)	.0162		
ALC <0.1 × 109/L	1.5773 (1244–2)	.0001			1.70 (1.12–2.54)	.0086		
ALC < 1 × 109/L	2.40 (1.32–4.14)	0004			2.16 (.95–5.04)	.0661		
ANC < 0.5 × 109/L	1.83 (1.13–2.93)	.0096			2.06 (1.27–3.92)	7		
Age ≥ 40 y	1.20 (0,87–1.69)	.26			1.13 (.73–1.85)	.59		
Active GvHD at the time of RVI	2.10 (1.15–3.87)	.01			2.73 (1.17–6.91)	0,02		
Recent allo-HCT or pre-engraftment	3.02 (.77–12.61)	107			2.05 (.29–.09)	.38		
Time from allo-HCT to influenza detection								
≤180 d	1		1		1			
181 to 365 d	.33 (.13–.80)	.01	.31 (.09–1.15)	.08	.51 (.14–1.67)	.27		
>365 d	.45 (.22–.93)	.03	.52 (.16–1.73)	.28	.64 (.25–1.67)	.36		
MAC	1.55 (.85–2.84)	.15			1.33 (.58–3.10)	.49		
Transplant period								
≥ 2020	.33 (.07–1.22)	.12			.29 (.01–1.66)	.25		
2017–2019	.52 (.24–1.07)	.08			.49 (.17–1.27)	.15		
2014–2016	.20 (.08–.44)	.0001			.28 (.08–.79)	.02		
< 2014	1				1			
Oseltamivir at the URTD stage	.75 (.32–1.61)	.48			.52 (.12–1.60)	.31		
Influenza vaccine^a^								
No	1		1		1		1	
Yes	.17(.05–.47)	.001	.18 (.04–.55)	.005	.22(.035–.8)	.04	.27(.04–1.05)	.09
Vaccination-ineligible	1.91(.79–4.63)	.14	.83 (.2–3.29)	.79	.47(.07–1.72)	.32	.24(.03–1.19)	.12
Coinfections	1.57 (.80–3.06)	.17			.92 (.32–2.32)	.88		
ISI								
Low risk (0–2)	1		1		1		1	
Moderate risk (3–6)	2.86 (1.44–5.97)	.0034	3.6 (1.65–8.34)	.0017	3.60 (1.28–12.83)	.02	3.46 (1.19–12.61)	.03
High risk (7–12)	26.5 (6.14–186.54)	.00008	24.9 (5.03–191.96)	.0003	14.28 (3.14–7.98)	.00063	26.74 (4.70–189)	.0003

Abbreviations: CI, confidence interval; Log. Regr, logistic regression model; OR, odds ratio; Cox Regr, Cox regression; HR, hazard ratio; ATG, antithymocyte globulin; Post-Cy, post-transplant cyclophosphamide; URTD, upper respiratory tract disease; LRTD, lower respiratory tract disease; GvHD, graft-versus-host disease; allo-HCT, allogeneic hematopoietic stem cell transplantation; mm URD, mismatch unrelated donor; Haplo id, haploidentical family donor; id sibling, identical sibling donor; id URD, identical unrelated donor; UCB, umbilical cord blood; MAC, myeloablative conditioning; CARV, community-acquired respiratory virus; ISI, immunodeficiency score index; ANC, absolute neutrophil count; ALC, absolute lymphocyte count; NT, not tested.

^a^A repeated multivariable analysis was performed excluding the vaccination-ineligible group, and influenza vaccination remained a significant protective factor against hospital admission (OR 0.18, 95% CI .045-.53, *P* = .0045) and showed a trend against oxygen requirement (OR 0.24, 95% CI .036-.9, *P* = .06).

For hospitalization, multivariable analysis identified the following independent risk factors: calcineurin inhibitor-based GvHD prophylaxis (OR 7.04; *P* = .01), high-dose (≥30 mg/day) corticosteroid use (OR 2.86; *P* = .04), moderate-risk ISI (OR 3.6; *P* = .0017), and high-risk ISI (OR 24.9; *P* = .0003). Again, influenza vaccination was protective (OR 0.18; 95% CI: 0.04–0.55; *P* = .005), as was donor/recipient HLA mismatch (OR 0.32; *P* = .02).

Regarding oxygen therapy, predictors identified in multivariable analysis were donor/recipient sex mismatch with female donor/male recipient (OR 3.24; *P* = .01), high-dose corticosteroid use (OR 8.21; *P* = .001), moderate-risk ISI (OR 3.46; *P* = .03) and high-risk ISI (OR 26.74; *P* = .0003).

### Causes of Death and OS

Overall, 10 patients (7%) died at a median of 37 days (range 8–91) after the last influenza episode. Causes of death were secondary malignancy (*n* = 1), viral infection (*n* = 3), interstitial pneumonitis (*n* = 1), invasive fungal infection (*n* = 1), bacterial infection (*n* = 2), hemorrhage (*n* = 1), and GVHD (*n* = 1). Infection and/or respiratory failure was the main cause of death in 6 patients, none of whom was vaccinated against influenza. Due to the limited number of events, no further statistical analyses were performed.

## DISCUSSION

The current study provides evidence supporting the clinical benefit of seasonal influenza vaccination in allo-HCT recipients and identifies additional factors influencing influenza infection severity in this population. We report an overall LRTD progression rate of 29% (combining possible and proven cases) in our cohort. Influenza vaccination showed an independent protective association against LRTD progression and hospital admission in multivariable analyses. Fever at the time of influenza screening was a useful surrogate marker of LRTD involvement, whereas the ISI was useful to predict progression to LRTD, hospital admission, and supplementary oxygen requirements.

The LRTD progression rate observed in our series aligns with the 13%–30% reported in prior studies [13, 21–23]. The key finding from the current study was the protective effect of influenza vaccination in reducing disease severity. Our group [13] and others [14] previously reported reduced transmissibility via a lower prevalence/incidence of influenza infection episodes in vaccinated allo-HCT recipients as compared with unvaccinated patients, but the impact on disease severity was not demonstrated. While our previous report indicated a trend toward reduced progression to LRTD (*P* = .05), we are now able to provide novel evidence that vaccination is associated with a significant reduction in disease severity. We observed a 72% decrease in LRTD progression, with rates dropping from 58% in unvaccinated to 16% in vaccinated recipients. Furthermore, vaccination significantly reduced the risk of hospitalization and showed a trend toward lower oxygen therapy requirements, thereby reinforcing its clinical benefit across multiple outcomes. In addition, the lack of significant differences in risk of LRTD progression between unvaccinated (58%) and vaccination-ineligible patients (37%) indicates that the interval elapsed from stem cell infusion to influenza infection alone is not enough to mitigate disease severity. The main difference between our previous report [13] and the current one is the larger number of influenza infection episodes in allo-HCT recipients included in the latter, which likely enhanced the statistical power to detect differences.

In the allo-HCT setting most vaccine clinical trials have traditionally used immune correlate of protection (ICP) as an acceptable surrogate endpoint for efficacy [24]. However, vaccinated allo-HCT recipients showed a poor seroconversion rate, ranging from 13% to 59% [25], and lower protective antibody titers compared with healthy controls [15, 16]. Although strategies to enhance immunogenicity such as high-dose vaccines [26], multidose schedules [27], multiple doses of high-dose compounds [28] or multiple doses with adjuvanted vaccine [29] have been explored (the latter 2 reaching >80% seroconversion), their clinical efficacy has not been demonstrated. The poor serological response and absence of clinical benefit may have diminished the perceived importance of vaccination. Many physicians might argue that the low probability of serological response in scenarios such as early transplant or moderate to severe GvHD leaves patients with a similar risk of severe infection regardless of vaccination. This may partly explain the historically low reported vaccination rates among allo-HCT recipients (<40%) [30–32]. In line with these numbers, only 25% of influenza episodes were vaccinated in our series (48 out of 190, excluding 24 vaccination-ineligible recipients). Although this figure cannot be interpreted as the overall vaccination rate in our cohort, since unvaccinated cases may be at higher risk of influenza transmission and may be overrepresented in this cohort, it remains remarkably low. Further epidemiologic studies are needed to determine the true vaccination rate in allo-HCT and thus enhance vaccination adherence policies in this population.

Despite the utmost importance of ICP, most vaccine clinical trials disregard T-cell immune response as a marker of vaccine response. Nonetheless, influenza vaccination was able to trigger peripheral blood T-cell activation, with CD4⁺ lymphocytes producing the Th1 cytokine IFN-γ, both in patients vaccinated more than 6 months post-transplant and in those immunized earlier [16]. The striking reduction in influenza severity achieved in our series with standard compounds, even in recipients with limited expected antibody responses (eg, <6 months post-allo-HSCT, presence of GVHD, ongoing immunosuppression or corticosteroid therapy; 52% of the vaccinated episodes in our series) suggests that influenza vaccination may also exert clinical benefit by eliciting cell-mediated immunity [33, 34]. This possibility, together with the favorable safety profile of the vaccine, opens the door to revisiting current guideline recommendations. Beyond adaptive immunity, influenza vaccination might confer protection in allo-HCT recipients through vaccine-induced trained innate immunity. Experimental data showed that certain vaccines can induce durable epigenetic and metabolic reprogramming of monocytes, macrophages, and NK cells, enhancing their antiviral responsiveness upon subsequent exposures [35]. In a setting where adaptive immune recovery is slow, such trained innate mechanisms may also contribute to the reduced disease severity observed in vaccinated patients. Our results support considering influenza vaccination at any time after transplant during influenza season and highlight the need to assess its potential clinical effectiveness in this patient subset through prospective clinical trials.

Regarding other predictors of disease severity, our results reaffirm the important role of the ISI in influenza virus infection severity. Although initially developed to predict progression to LRTD and mortality in respiratory syncytial virus, the ISI has also been validated for influenza [13, 36]. In our cohort, it proved a robust tool for identifying patients at higher risk of progression to LRTD, hospitalization, and oxygen support, underscoring its value in clinical decision-making. Additionally, fever at the time of CARV screening showed good discriminative ability for anticipating LRTD and could serve as a trigger for radiological assessment in these patients. Future research should explore whether incorporating factors such as vaccination status and fever at CARV screening could further enhance the predictive performance of the ISI.

Other factors associated with hospitalization and oxygen requirement in our cohort were closely related to a higher degree of immunosuppression. These included female donor/male recipient transplants [37] and calcineurin inhibitor-based GvHD prophylaxis [38], both linked to increased risk of GvHD. This scenario often necessitates prolonged and continuous administration of corticosteroids and/or other immunosuppressive agents to manage GvHD, which in turn sustains a chronic state of immunosuppression and increases susceptibility to severe CARV infections. Indeed, corticosteroid use, known to impair T-cell-mediated responses against viruses [39], was also associated with severe influenza virus disease. Although donor–recipient HLA mismatch has traditionally been associated with a higher risk of GvHD and mortality [40], in our series this condition appeared to have a protective effect against hospital admission. This unexpected finding may be partially explained by the lower rates of corticosteroid use (32% vs 46%, *P* = .06) and female donor/male recipient transplants (15% vs 30%, *P* = .02) within the HLA mismatch group.

We acknowledge several limitations of the current study, including the retrospective observational design, relatively small sample size and somewhat heterogeneous vaccination policy. Although the performance of the different diagnostic platforms used in the current study were comparable, minor differences in sensitivity and specificity between assays exist and may have introduced some variability in virus detection. The precise timing of progression from URTD to LRTD could not be accurately determined, as radiological confirmation was used as the reference point; subtle early lower-tract involvement may have occurred earlier but cannot be reliably identified in real-life clinical practice. We also cannot exclude residual confounding related to preventive behaviors, as patients who choose to be vaccinated may also adopt additional protective measures (eg, mask use and crowd avoidance), which were not captured in our dataset and may have influenced the risk of LRTD. Nonetheless, our study spanned 10 consecutive influenza seasons, thus minimizing potential bias from year-to-year fluctuations between vaccination coverage and circulant influenza strains, and was supported by data from our prospective CARV registry, both of which should be regarded as major strengths.

In conclusion, our data provide real-world clinical evidence of the key role of influenza vaccination in reducing disease severity in the allo-HCT recipients. Additionally, we confirmed the valuable role of the ISI for risk stratification in allo-HCT recipients with influenza infection. Expanding influenza vaccination coverage in this high-risk population should be a priority.
